# The Hospital Nursing Supplement

**Published:** 1896-02-15

**Authors:** 


"The Hospital" Nursing Mirror.

Being the Extra Nursing Supplement of "The Hospital" Newspaper.

[Contributions for this Supplement should be addressed to the Editor, The Hospital, 428, Strand, London, W.C., and should have the word "Nursing" plainly written in left-hand top corner of the envelope.]

### News from the Nursing World.

### INCURABLE CASES AT WESTMINSTER.

The nursing of chronic and incurable patients is apt to be considered as a doleful and depressing duty by those who have no experience of the work. Such an opinion cannot long be maintained by anyone entering the ward at Westminster Hospital which is devoted to this class of patients, for it is one of the brightest spots in the house. There are plenty of windows, and they command a view of the Abbey, St. Margaret's Church, the Houses of Parliament, and, by no means the least attraction to the patients, a pleasant prospect of the street life, with its constant traffic, from which infirmity has cut them off. The ward is fresh and bright, and, strange to say, so are the patients. Always helpless, and often suffering, as some of them are, they seem cheerful and comfortable. The secret of their contentment lies not only in the patient nursing and skilled care given to poor afflicted bodies, but in the pleasant and varied occupations provided. With undisguised pride these women and girls dress dolls by the dozen in the winter, and when a sufficient number have been made ready for the patients at Westminster they then busy themselves with the preparation of equally pretty gifts for children elsewhere. The dolls are charming, and so are their dainty little garments, but there is a deeper attraction about these offerings made by the incurables for those poorer than themselves. Excellent pinafores for their own wear are made in this Westminster ward by the girls, and many other useful things. Just now, to give a change of occupation, the matron, to whom so much of their happiness is due, has introduced knitting for the Deep Sea Fishermen, and it is pleasant to see delicate (and, alas! often distorted) fingers busy with the warm gloves and vests which will be gratefully accepted by the active toilers in the North Sea.

### WORKHOUSE INFIRMARY NURSING ASSOCIATION.

The Workhouse Infirmary Nursing Association inserts in its latest annual report a resolution of the executive committee of so reasonable a character that it ought nowhere to meet with opposition. It runs as follows: "That in view of the very considerable expense incurred by the committee in training nurses, Boards of Guardians are expected to subscribe to the funds of the association in the proportion of one guinea annually per nurse's place kept filled by the association." The provision of trained nurses for workhouse infirmaries is the object for which the association was first established in 1879. It arranges and pays for the training of probationers, and provides for systematic visiting of nurses in country workhouses. This friendly supervision is much valued by workers, who are often very much isolated, and it is pleasant to read of the cordial recognition of their work which is reported to the association by workhouse masters, Guardians, and patients. The demand for the association nurses is an ever-increasing one, but lack of funds limits the supply, and subscriptions are urgently needed for training more probationers. All particulars of the work can be obtained from the office, 6, Adam Street, Strand, and donations will be gladly received and acknowledged by the hon. secretary, Miss Wilson, 13, Barkiston Mansions, S.W.

### NURSING GUILD AT RICHMOND.

The annual meeting of the St. John Ambulance Nursing Guild at Richmond took place last week. It was held at the house of the president, the Hon. Miss Brodrick, and there was a large attendance, the chair being taken by Colonel Sparks. A trained district nurse is employed by the guild, and she is assisted by the lady associates, who visit the sick and distribute gifts, and make loans of nursing appliances. In the course of last year 234 sick persons received 4,284 visits. The visits paid in 1894 were reported as 2,500; the work of the guild, therefore, appears to be increasing very rapidly, and receives the countenance and support of the local medical men.

### GOVERNMENT RECOGNITION OF GOOD NURSES.

The nursing staff at the Government Civil Hospital, Hong Kong, were agreeably surprised on Christmas morning by the receipt of a most beautiful tea and coffee service. This presentation was made by Government, and was accompanied by a letter which stated that the services rendered by the Sisters last year during the plague visitation at Hong Kong had been brought to the notice of the Secretary of State. This handsome gift is therefore a public recognition of the aid then rendered by them. The various pieces are beautifully chased, and the stand has a shield on either side, one bearing an inscription stating the presentation was made by Government, and the other engraved with the names of the Sisters, the majority of whom were trained at the London Hospital: Miss C. Eastmond (matron), E. G. Ireland, C. McIntosh, E. F. Heggin, A. Brookes, C. Walker, E. M. Palmer, A. Penruddocke, S. E. Barker. Previous to this official presentation, the nursing staff received from the residents at Hong Kong a handsome silver centre-piece for the dinner-table, and medals for themselves, as a testimony of valuable services to the community at a time of extreme peril.

### QUEEN'S NURSES AT TUNBRIDGE.

The attendance at the annual meeting of the Tunbridge Wells District Nursing Association showed the widespread interest taken in its work. The chairman spoke in high terms of the valuable advice and assistance received from the Queen's Jubilee Institute, with which the association is affiliated. Reference was also made to the excellent rule which provides for the duties of the nurses being carried on under medical control. Three nurses reside in the Home which was provided for them last year, and the Queen's Inspector has given a favourable report of their work and of the condition of the Home.

### A GENEROUS GIFT.

A most opportune gift of £3,000 has been made by Mrs. C. E. Lees for the erection of a Home for the Oldham district nurses, who sadly need proper headquarters. Their services are highly valued in Oldham. Many whom they have successfully nursed will be glad to learn that the erection of a comfortable permanent establishment will soon be proceeded with.

### LAUNDRESS OR NURSE.

The Congleton Guardians have been advised by the Local Government Board Inspector to appoint a second nurse, as he considered that there was too much work for one, there being a number of cases of pneumonia in the wards, which no one woman could possibly look after night and day. The advice given by the inspector was duly considered at a recent board meeting, and (according to the local press) some original views were shown to prevail at Congleton with regard to the qualifications of an assistant nurse. It was decided that she must combine the duties of seamstress with those of assistant nurse, and it is stated that, after a short discussion, the Guardians agreed that the present laundress at the workhouse should be promoted to the composite post. Of course, this woman may be a trained nurse, although it hardly seems probable, and her qualifications are not alluded to in the report of the meeting. The patients, whose condition caused the inspector to recommend the additional nurse, are not likely to receive much benefit from the intermittent services of an ex-laundress and present seamstress. It is improbable that such an appointment will receive the sanction of the Local Government Board, which holds a somewhat higher standard of a nurse's qualifications than the Congleton Guardians.

### THE MIDWIVES BILL.

At a large and influential meeting recently held in the Co-operative Hall at Leicester, some interesting details on the subject of midwives and their work were given by the various speakers. It was stated that in one year alone six thousand women had died in childbirth in England and Wales, the majority having been attended by ignorant women. Mrs. Creighton, who presided, made an able speech, in which she said, "the health of the community was the concern of the community, and the health of the wives and mothers was of the greatest importance to all." Other speakers followed, and it was pointed out by one of the doctors present that the proposed Bill for Compulsory Registration of Midwives was essentially a woman's question, and one which all women should help to advance. Those present were unanimous in appreciating the fact that the Bill was not a matter of doctors *versus* midwives, but was rather designed to secure for the poor women of England duly trained instead of ignorant attendants. Marked attention was paid by the audience to an interesting address given by Miss Rosalind Paget, member of the Midwives' Institute, London, and herself a trained nurse. Miss Paget traced the history of midwives and their position in the past and present, explaining that 70 per cent. of the births amongst the working classes took place in the absence of a medical man.

### IRISH DISTRESSED LADIES' FUND.

The work of the society which bears the title of Irish Distressed Ladies' Fund is more varied than its name implies. Not only does it give pensions to the infirm and aged, and grants to relieve urgent suffering and distress, but it helps gentlewomen of reduced means to dispose of their needlework, and it secures education for the young. Two girls have been trained as technical teachers in cookery, laundry-work, and dressmaking, and both have obtained diplomas and subsequent engagements. This most practical education is certainly not the least useful part of the work of a society of which Her Majesty the Queen is patron and Princess Louise president. The offices are at 17, North Audley Street.

### WREXHAM DISTRICT NURSING SOCIETY.

A year ago the Nonconformists at Wrexham decided to establish a trained district nurse in the town. The movement was well supported, and the society, in presenting its first annual report, shows a creditable balance in hand. Nurse Wilson attends persons of all creeds, has paid 2,000 visits to 73 patients, and her services and instructions have been gratefully received in all quarters.

### THE COMPANY OF PUBLIC SUCCOUR.

An interesting account is given of the branch of the "Military Brotherhood" at Florence known as the Company of Public Succour, of which Queen Margaret is patron. The brotherhood was organised in 1878 by King Victor Emanuel, and received the title of "Fratellanza Militare," and is formed of retired soldiers of good character. The Company of Public Succour consists of 150 members, who voluntarily bestow their services on any who require them. Aid Committees have been established, and the associates observe military discipline. They attend the sick in their homes or transport them to hospitals when required, sometimes going quite long distances in emergencies. They are strictly forbidden to accept any payment for their services. As a medical contemporary says, "it is sufficient to see them handle a patient to know that they are adapted both by nature and training to the work."

### SHORT ITEMS.

A meeting has been held at Girvan to consider the question of a trained district nurse being engaged for the town and neighbourhood.—Nurse Thurlow has resigned her post at Yarmouth Workhouse after twenty years' service. A superannuation allowance is proposed for her, and it is hoped that a trained and experienced nurse will be secured for the vacancy.—Nurse Salter's services appear to be highly appreciated at Ashburton, where she holds the position of district nurse.—The erection of a fever hospital at Belfast is contemplated, and attention has been called to the urgent need of accommodation for the typhoid patients, who are far too numerous for the district nurses to undertake to look after them in their own homes, even if such an arrangement were desirable.—At Woodborough the guardians accompanied their consent to the payment of an annual subscription to the local district nursing association with remarks on the value of the services rendered to persons who in many cases were kept off the rates by timely aid.—A successful concert was given at Redditch for the benefit of the Nursing Association.

### Lectures on Nursing.

By a Superintendent of Nurses.

### X.—FOOD.

One of the things which distinguish an animate object like the human body from an inanimate one is, that in the former there is constant wasting away of its parts, which waste has to be replaced by new material, and this new material we call food. The food-stuffs are divided into two great classes, the organic and inorganic. The organic includes proteids, fats, and carbohydrates; the inorganic, water and mineral substances, and particularly salts of sodium, calcium, and iron.

Proteids.—The common examples of these in the animal kingdom are white of egg and the lean of meat; while in the vegetable kingdom we get legumen in peas, beans, and the cereals like wheat. The proteid-containing foods are broken up small in the mouth by mastication, but are not absorbed until they reach the stomach, where the gastric juice, containing pepsin and hydrochloric acid, changes them into peptone, a substance which is soluble and can pass into the capillaries, or small blood-vessels of the stomach. Any proteids which are not absorbed in the stomach are acted upon by the pancreatic juice.

Fats, which supply heat, are principally, but not entirely, furnished by the animal kingdom, and undergo no change, except being broken up in the mouth, until they reach the duodenum (the upper part of the small intestine), where the bile emulsifies them, separating them into the tiniest particles, and the pancreatic juice converts them into a soap-like substance. They are then absorbed or sucked in by the lacteals, which are vessels that convey the chyle or nutritious part of the food from the intestines to the thoracic duct. This is a small vessel that opens into one of the large veins of the left side of the neck.

Carbohydrates are of two kinds, starches and sugars. Starches are found chiefly in roots and seeds. They are less easily digested than sugar, because before they can be assimilated or absorbed by the system they have to be converted into sugars. This is effected partly in the mouth by the action of the saliva and partly in the intestines by the pancreatic juice. On the other hand, the sugars after having been converted into grape sugar are easily absorbed into the blood. It should be remembered that the saliva of very young children does not possess the power of converting starch into sugar. Hence the importance of not giving them starchy goods like arrowroot or potatoes.

Salts are absorbed into the blood at once, either in the mouth or stomach through the thin walls of the capillaries. Salts of iron deserve special mention seeing that the red corpuscles of blood contain a large percentage of iron and probably owe their oxygen-carrying powers to its presence. It is to the presence of calcium that the hard parts of the body, such as the bones and the teeth, owe their properties. Mothers therefore should see that their children have a plentiful supply of whole meal bread and porridge, because in the milling processes which convert the grain into the "purest whites" a large proportion of the calcium salts is lost.

Water is absorbed more or less from all parts of the digestive track. It plays an essential, if not the most essential, part in the nourishment of the system. There is not a single tissue in the whole body into the composition of which water does not enter. It forms 70 per cent. of the whole weight of the body, and all the nutritious substances are without exception finally carried to the tissues of the body, dissolved, or suspended in water.

Not only is this the case, but water is the fluid by means of which all waste products are carried off. First, by perspiration. It has been estimated that from two to three pounds of water are daily evaporated from the skin, and this is hardly to be wondered at when one learns that there are no less than 28 miles of tubelike sweat glands on the surface of the human body. It is also thrown off from the lungs when we breathe, and lastly, a large quantity is excreted by the kidneys. By means of evaporation of the sweat, too, the temperature of the body is regulated.

To realize how important an article of diet it is, one has only to recall to mind the cases of the men like Succi, who have lived for fifty or even more days without any other form of food than water.

A person doing an ordinary amount of work would require per day of proteids, 3½ oz.; of fats, 3½ oz.; of carbohydrates, 8 oz.; of water, 80 oz.; of salts, 1 oz. But it has been found that the mere presence of these food stuffs in the above quantities and proportion in a diet is not of itself sufficient to ensure good health. A certain proportion of the food must be fresh. It is owing to a want of appreciation of this fact that so many sailors have in past time lost their lives at sea from scurvy, and that nowadays so many little children, if not actually losing their lives, suffer from ill-health.

Milk as an article of diet deserves special mention, as not only does it contain all the various food stuffs in the proper propertions, but it has antiscorbutic properties, *i.e.*, properties that safeguard against scurvy.

But in settling a diet, especially when doing so for a sick person, we must consider not merely its chemical composition, and whether or not it possesses anti-scorbutic properties, but also whether it can be easily digested. For instance, cheese and cold hard-boiled eggs, considered from a chemical point of view, are excellent articles of diet, and, indeed, for healthy individuals starting out for a day's picnicking to be highly recommended, but they are hardly articles of diet for the sick chamber.

We may briefly summarise the changes that food undergoes in its passage from the mouth to the blood-vessels which convey the nourishment to all parts of the body as follows: It is ground fine by the teeth and being moved about by the tongue is mixed with the saliva. The saliva not only moistens the food but mixes up with it the substance, ptyalin, which converts its starch into sugar. The saliva is secreted in glands which open into the interior of the mouth, and discharge a liquid varying in quantity in a full-grown person from 8 oz. to 24 oz. in the twenty-four hours. It consists principally of water, but a small quantity of saline matter is mixed with the ptyalin.

It is therefore most necessary that the food should not be "bolted," but that people should thoroughly masticate all they eat. On reaching the stomach the food is thoroughly mixed with the gastric juice, and by it has part of its proteid materials converted into soluble peptones.

After this process the semi-solid acid food, now called chyme, passes on to the upper part of the small intestines. Here its presence causes an active secretion of bile from the liver, and of an alkaline juice from the pancreas. The bile, as before stated, emulsifies the fats, while the action of the pancreas is threefold.

(1) More insoluble proteids which have escaped the action of the gastric juice are now made soluble.

(2) The fats are saponified.

(3) Carbohydrates, which have escaped the action of the saliva, are converted into grape sugar.

The food now is in a condition to have its soluble parts absorbed by the lower part of the small intestines. This is effected partly by the lacteals on its walls and partly by the blood-containing capillaries. In both cases the ultimate destination is the heart, but that portion taken up by the capillaries passes by way of the liver to the heart, whilst that absorbed by the lacteals is conveyed by the thoracic duct to the veins of the neck and so to the heart.

From the right side of the heart the blood is pumped into the lungs, to be aerated, whence it returns, bright red in colour, to the left side of the heart, and is then distributed to the hungry and thirsty tissues of the body.

### Nurses' Co-operation.

### ANNUAL MEETING.

The business before the ordinary general meeting of the members of the Nurses' Co-operation, which was held at 8, New Cavendish Street, on Tuesday last, was of a purely formal character. Resolutions were passed re-electing Mr. Michelli and Miss Gethen as members of the committee of management, the auditors for the ensuing year were re-elected, and the accounts, balance-sheet, and annual report were passed.

At the nurses' meeting on February 7th Mr. Bickersteth and Nurse Slater were re-elected nurse representatives.

The report for 1895 shows the society to be not only continuing but increasing in prosperity, the gross receipts for the year amounting to £27,389 19s. 1d. (including fees outstanding on December 31st), as compared with £23,218 18s. 11d. and £18,762 11s. 4d. in the years 1894 and 1893 respectively. The income exceeded expenditure in the past year to the amount of £824 2s. 4d., the capital of the Co-operation now standing at £2,023 19s. 11d., the greater part of which sum is invested. A sum of £24,228 7s. 10d. was paid over to the nurses during the year, and the income of the society, derived from the 7½ per cent. deducted from the total earnings of the nurses, together with £56 10s. 5d. received in home fees and £24 11s. interest on investments, amounted to £2,050 9s. 7d.

That the Nurses' Co-operation is popular is proved by the number of applications, an average of 400 having been received at the office previous to each half-yearly election, and this though nurses have not been advertised for. It is felt by the committee that the almost unprecedented success of the Co-operation is doubtless in large measure due to the wise administration of Miss K. Philippa Hicks, the lady superintendent, who, "in addition to her indefatigable devotion to her own department, has given much aid to the committee in forwarding the interests of the nurses and in developing the work of the society." The committee of management specially note in their report the high standard of training and efficiency which characterises most of the candidates who apply for election on the staff, a fact which testifies to the estimation in which the Co-operation is held in the nursing world. Beginning with a roll of 185 nurses in 1891, the number has risen with each year to 333 at the present time, and on the very day of the meeting Miss Hicks reported 307 of these as being in actual work. Nurses have been sent during the year to all parts of the world, including America, Africa, Japan, Egypt, and many parts of the Continent. During 1895 a circular was issued to the nurses on the staff, pointing out the need for making provision for old age, and explaining that the Co-operation paying the nurses the whole of their earnings (less 7½ per cent. for working expenses) the committee could not hold themselves responsible in case of disablement from age or infirmity, and directing attention to the encouragement to thrift offered by the Royal National Pension Fund for Nurses. Steps are to be taken very shortly to establish a nurses' home in connection with, and near, the office in New Cavendish Street.

### Nursing in Canada.

### GRADUATED NURSES AT THE TORONTO GENERAL HOSPITAL.

The annual graduating exercises of the Training School for Nurses were held on January 23rd in the amphitheatre of the General Hospital.

The event was made the occasion of a pleasant gathering, there being present many friends of the graduates, in addition to the house staff and hospital workers.

Mr. W. S. Lee, the chairman, briefly addressed the graduates, after which the following programme was carried out: Recitation, Miss Forbes; reading, Rev. Arthur Baldwin; song, Mr. J. Baker; address, Dr. I. H. Cameron; report for the year, Miss M. A. Snively, superintendent of the school; address, Dr. Parkin; song, Miss Louisa Craig. The certificates were presented by Dr. Charles O'Reilly, medical superintendent of the hospital, who reminded the graduates that the certificate of the school was in each case granted conditionally, and that its return might be demanded should the recipient be guilty of conduct unbecoming either a trained nurse, a true woman, or a lady, or should she betray the confidence of her patients or patrons, or be negligent, careless, or indifferent in the performance of duty.

Following is a list of the recipients of certificates and badges: Misses Mima Gordon, Orillia; Mary Burt, Hillsburgh; Jessie M. Porte, Toronto Junction; Annie Brown, Grahamsville; Emma Hall, London; Matilda Craig, Kingston; Annie I. Brown, Gravenhurst; Annie Lennox, Toronto; Florence N. Davies, Cayuga; Harriet Thompson, Malvern; Ella A. Wood, Toronto; Emma Parmenter, Toronto; Mary C. Stewart, Britannia; Sarah E. Bliss, Compton, Que.; Ethel A. Bayly, Toronto; Adela A. Drew, Oshawa; Jessie Christie, Toronto; Helen Flett, Mount Forest; Lucy Bowerman, Napanee; Virginia M. Taylor, Collingwood; Margaret M. Campbell, Toronto; Elizabeth G. Flaws, Toronto; Bridget Kennedy, Toronto.

### St. John Ambulance Association Nursing Corps.

A lecture was given at Ipswich on the 11th inst., under the auspices of the local branch of the St. John Ambulance Association, entitled "Good Nursing, and How to Get It." There was a good attendance in the Co-operation Hall, the chair being taken by Mrs. Bartlet in the unavoidable absence of the energetic hon. secretary, Miss Coulcher. In introducing the lecturer, Miss Gethen (formerly Sister Queen, of the London Hospital), Mrs. Bartlet alluded to the regret which all the members must feel at the absence on this occasion of one who had done so much to advance the interests of the Association. As mayoress Mrs. Bartlet won golden opinions last year from the members of the Royal British Association so courteously entertained by Dr. Bartlet in the course of a week which will be long remembered in Ipswich; and the lady certainly proved herself as able as a chairman as she had previously been hostess. The lecture received marked attention, and at its conclusion a cordial vote of thanks was passed to Miss Gethen for her practical advice and sound teaching as regards nursing, which must prove useful to every woman in the world at some time or other in her life.

### Minor Appointments.

Pendlebury Children's Hospital.—Miss Cecil Bennett has been appointed Sister-in-charge of the fever block attached to this hospital. Miss Bennett has had much experience in general nursing and fever work, and has recently held the post of charge nurse at the North-Eastern Hospital, Tottenham.

### Our American Letter.

A GREAT work has been done during the past year by the New York City Society, at the Lying-in Hospital, the annual report stating that seven thousand destitute women were cared for there. Some very liberal contributions have been made to the funds, and one friend has defrayed the whole cost of the much-needed and extensive alterations which have been recently completed. The hospital is a handsome building, and is now thoroughly equipped, and it is intended to largely extend its sphere of usefulness by establishing small branch stations in the poorer localities.

An anonymous friend has offered to undertake the cost of building an additional block at the Pennsylvania Hospital, Philadelphia, which will, it is estimated, require 100,000 dols.

At Greensburg, Pa., the Westmoreland Hospital has been remodelled and refitted, and appears to have every prospect of a successful career under the able medical superintendence of Dr. Frank Gowan, who has a valuable ally in Miss Clark, the matron, a thoroughly trained nurse from the West Penn. Hospital.

Great interest has been taken in the departure for Armenia of Miss Clara Barton, president of the American National Red Cross Society. She has gone to Constantinople to distribute relief to the Armenians, if she can succeed in reaching them. Miss Barton, who is about sixty years of age, has taken with her a secretary, Mr. G. H. Pullman, a stenographer, Miss L. Graves, and is accompanied by Dr. J. B. Hubbell, general field agent of the Red Cross Society, and Mr. Ernest Mason. Should this party succeed in carrying out their plan they will be followed by a party of American nurses.

The matron of the Philadelphia Polyclinic has established a system of post-graduate training at that institution. It commenced in May last, and four nurses have received diplomas at the conclusion of a six months' course. If applicants come direct from their training school a reference is required from their late superintendent. From those nurses who have been engaged for some time with private cases, references must be also given to the doctors under whom they have worked. No one is admitted to the post-graduate course who cannot show satisfactory antecedents, nor are those eligible who have failed to obtain the diploma of their training school. Miss Banfield (matron of this Philadelphia Polyclinic) does not admit the post-graduates for less than six months. No payment is given for their services beyond board, lodging, and laundry. They have, in addition to the course of general nursing, a course of housekeeping, if desired; also cooking, operating-room work, and massage or electricity.

The gold medal presented to the graduates at the Massachusetts Clinton Hospital has a strangely familiar appearance to the eyes of English nurses, for it bears the motto of the Prince of Wales, the words *Ich Dien* being engraved on a cross on one side, the recipient's name adorning the reverse side.

In spite of opposition and discouragement the American Pension Fund for Nurses is making quiet and steady progress in Philadelphia, and a bazaar, organised by the promoters of the scheme in December, resulted in a clear profit of 1,200 dollars for the new society.

### Nurses' Quarters at Bristol.

AN extension of the Nurses' Home at Bristol General Hospital is a scheme which appears to be regarded with considerable favour in the town. An offer from a member of the committee of a sum equal to that given by each of three other donors has been already responded to by one promise of £500 and another of £100. Doubtless many other liberal donations will follow.

### A Riviera Nursing Home.

### VILLA D'ORMESSON, CANNES.

THE Villa d'Ormesson was built by the Count of that name in 1878, and was duly fitted up with every comfort and modern convenience as a winter home for himself. Since 1892 it has been rented by Mrs. Dan Norris, an English trained nurse, with the idea of supplying some place which, while possessing all the comforts and conveniences of an hotel or well-appointed private house, might be more acceptable to many invalids as a temporary residence than either the more expensive alternative of renting an entire house, or the publicity of an hotel, also providing, if needed, the best sick nursing.

The villa occupies a good site near the western end of Cannes, on the southern slope of the Croix-des-Gardes Hill, and possesses a garden of its own of nearly three acres. Though only four hundred yards from the sea, the house stands 180 feet above it, owing to the sharp slope of the ground, and has consequently grand views from its windows, looking seawards over to the Isles de Lérins, and westward over La Bocca, the Bay of Napoule, with a foreground of terraced gardens and pine-covered hillsides, and the Estérel Mountains in the distance.

The sanitary and heating arrangements are on the English system, and water is laid on to every floor. The principal bedrooms are ten in number, with others for nurses and servants, and there is also an annexe, with stable, coach-house, and additional bed-rooms.

Mrs. Norris has herself had exceptional nursing experience. She was trained at St. Thomas's Hospital, afterwards being assistant lady superintendent of the Royal Infirmary, Edinburgh, and subsequently for nine years matron at St. Mary's Hospital, Paddington. Besides this, Mrs. Norris was selected by the Government at the time of the war in the Soudan to take charge of the Royal Victoria Military Hospital at Suez, and for her services there received on her return to England the Order of the Royal Red Cross. She is also known in the English nursing world by her "Notes on Nursing." A staff of carefully selected nurses is maintained under Mrs. Norris's directions for work in Cannes throughout the season.

Invalids ordered to the Riviera for the winter and seeking the quiet of home life combined with skilled care in case of serious illness thankfully welcome the opportunity thus afforded, and they are, of course, at liberty to choose their own medical man. The villa is primarily for the benefit of invalids, but in certain cases room can also be found for friends or relatives. Inquiries respecting terms, which are inclusive except for special nursing, wine, and bed-room fires, should go direct to Mrs. Dan Norris, in the winter at the Villa d'Ormesson, in the summer at 1A, Dennington Park Mansions, West Hampstead, N.W.

### Chicago Woman's Club.

THE Chicago Woman's Club has just issued, as its "nineteenth annual announcement," the report of 1895-6. Besides the usual officers, there is a board of twenty-four directors, a committee of management, and various other standing committees. The club was incorporated in 1885, has a large number of members, its rules appear to be of a practical character, and its objects are stated to be "mutual sympathy and counsel, a united effort towards the higher civilisation of humanity, and general philanthropic and literary work."

### Women Workers in New England Hospitals.

### NEW ENGLAND HOSPITAL FOR WOMEN AND CHILDREN, BOSTON.

THE story of this hospital is specially interesting as showing what is being done by women on the other side of the Atlantic for the help and benefit of their own sex. Founded so long ago as 1863 by three ladies, Miss Lucy Goddard, Dr. Marie E. Zakrzewska, and Mrs. Ednah D. Cheney, its objects were to be: (1) to provide for women medical aid by competent physicians of their own sex; (2) to assist educated women in the practical study of medicine; and (3) to train nurses for the sick. To these aims the hospital has remained faithful, and to-day not only is the medical staff composed, with the exception of the consultants, wholly of women, even to the dental surgeon, but the governing body also numbers more women than men among its members.

While possessing the proud distinction of having been the first in the United States to start a Training School for Nurses, in the development of district nursing this New England Hospital has also taken a pioneer part, and the dispensary attached to it in one of the poorest quarters of Boston is an important factor in the training of both its internes and nurses. A service of three months at the dispensary is required of the internes, and there is also a resident dispensary physician, so that sudden calls can be promptly responded to, and the visiting among the poor carried on with thoroughness. The valuable help thus afforded to the women of the lower classes in Boston is very greatly appreciated, and none the less so that all patients able to afford it are charged a fee of ten cents. That this payment is gladly and willingly made is evident from the report of the medical staff, who state that there are many "self-respecting, hard-working women who are obliged to make two visits each weeks for months in the gynæcological department, who never fail to bring their little fee, but who could not afford to go to a doctor's office for the prolonged treatment necessary in such cases." The dispensary patients pay twenty-five cents each upon their prescriptions, except in those cases where investigation proves inability to pay, when it is at the discretion of the physician to give free tickets. The dispensing is presided over by a lady pharmacist.

The custom which has prevailed for some years past at various diet kitchens and similar institutions in the district of supplementing the medical work of the hospital by allowing the physicians to distribute free cards for the nourishing food there supplied, and undertaking the feeding of sick children and babies at nominal prices, is a practical charity of the highest value. All who have ever worked among the sick poor know well the uselessness of medicines when the poverty of the patients means that proper food is unattainable.

Patients of all nationalities are treated at the dispensary, and admitted into the hospital. The growth of the dispensary work has made new and larger buildings imperatively necessary, and this it is the aim of the directors to accomplish as soon as possible—in other words, as soon as funds will allow.

The Training School for Nurses does its work quietly and smoothly in charge of the superintendent of nurses, and under the supervision of the resident physician. There is a staff of twenty nurses; the period of training has just lately been extended from eighteen months to two years. The addition of a new surgical building, which is hoped for, will give the nurses experience in fever cases which cannot now be admitted owing to want of sufficient accommodation, and the impossibility of treating such cases under the same roof with surgical patients. A development in private nursing is also contemplated in the near future. The education of the probationers is carefully looked after, and very complete courses of lectures are given by the various members of the medical staff. The syllabus for the present year began with a lecture by Dr. Marie E. Zakrzewska on the "Relation of Nurse to Physician, Patient, and Families of Patients," followed by systematic instruction in anatomy, physiology, surgical, obstetrical, and medical nursing, and concluding with the treatment of special subjects. The lecturing physicians are all women.

It is disappointing that subscriptions and donations should have decreased of late years, being only 42 per cent. of the amount obtained from these sources in 1882. Board and treatment receipts and donations for yearly free beds were considerably less in 1895 than in the preceding year, while the decrease in income from invested funds was much less marked. "All this," states the treasurer's report, "means two things: First, a tendency—and a growing tendency—toward becoming an endowed institution instead of living from the contributions of those giving each year. This is common to many successful institutions, though dangerous in the end, perhaps, in diminished stimulus. Second, it means that a very large part of our work is given in charity." Invested property amounts to something over £100,000. The annual income is insufficient for necessary expenses, and this deficit would be met if the subscription list were doubled. The state of matters deplored by the treasurer means that, in fact, the present generation is failing to recognise its responsibilities by not adequately supporting the institutions which minister efficiently to its needs. The women of Boston might see to it that this particular hospital, which does so much for their sex, shall not be crippled in its useful work for want of money.

### Doll Show and Competition.

WITH the praiseworthy intention of assisting towards the salary of the district nurse, some energetic and helpful inhabitants of a large and very poor Wiltshire parish are organising a doll show for Easter Monday. It is also a competition, for prizes are offered for the best-dressed doll. Many people take up some extra charitable work during Lent, and here is an opportunity for some who may have a little leisure to devote to such an object. They will have the satisfaction of feeling that they are helping to forward the cause of true charity, for no more real kindness can be done to the poor of any district than to provide them with skilled care and attention in times of illness. The hon. secretary of the scheme, Mrs. Ribbans, Sherston Vicarage, Malmesbury, will supply full information to all inquirers.

### Appointments.

BANGOR INFECTIOUS DISEASES HOSPITAL.—Mrs. Rice, who was trained at the Adelaide Hospital, Dublin, has been appointed Matron of the Infectious Diseases Hospital, Bangor. We congratulate her on her promotion.

ST. MONICA'S HOSPITAL, EASINGWOLD.—Miss Evelyn A. Mansel has been made Nurse-Matron of this hospital. She was trained at Charing Cross Hospital, afterwards worked as a Queen's Nurse for two and a half years, and then went to Guy's Hospital. We wish Miss Mansel success in her new work.

### On Certain Aspects of the Nursing Question as Seen in England and Germany.

By a Certificated Midwife.

### I.—INTRODUCTORY.

### England and Her Ways.

The system of nursing in England has wonderfully developed and improved within the last thirty years, save in one branch, which still stands in great need of regulated extension and practical force. I allude, as my readers will guess, to the nursing of women in childbirth. Here much remains to be done, and I have been asked to tell what I know of the difference between England and Germany as regards the training of those women who take up midwifery, and what is commonly called monthly nursing. To give my words any value it must first be stated that I have been trained as midwife and monthly nurse in both countries, and hold certificates from both. I was also trained in medical and surgical work in an English general hospital, and was a patient in a German one for eight weeks, so that I am in a position to compare the methods of the two countries from personal experience, which is an unusual advantage.

In drawing deductions from my experience I do not wish to lose sight of the fact that with all our casual—and I might say slovenly—methods, we retain the inestimable blessings of liberty of action, and I will candidly confess at once that I do not see how we are to combine the discipline, thorough training, and economy of the German schools of midwifery with the pleasant freedom from State interference which we now enjoy. But wiser heads than mine are giving their best attention to this subject, and all I shall attempt is to set before my readers the different practices of the two nations as far as I have grasped them, and by this means make it easier for the general public to see the bearing of the question.

There is no doubt whatever on the preliminary point, that all women, whether rich or poor, require to be attended to and taken special care of during childbirth. From the Queen on her throne, down through all grades of society, the mother in her necessary suffering, possible danger, and temporary helplessness appeals for aid. On the efficiency of this aid depends the welfare both of mother and child, and on the quality of this nursing is based the health and strength of the men and women of the land. In uncivilised countries the women fare better in childbirth, being more in a state of nature as regards animal conditions of life, and so get through their trouble with but little danger, as do brute beasts. But we are a civilised people and thank heaven for it, though we must take the burdens of civilisation up with its blessings; and one of its burdens is the attendant pain and peril of bringing forth offspring. Since it is our social and corporate life which accentuates this burden, the community (in all lands) acknowledges the duty of mitigating its evil as far as may be; in one country by private enterprise, public benevolence, or individual effort; in another by State regulations. Further, we all know that the nation which succeeds best in taking care of its mothers will produce the finest race of warriors, thinkers, and labourers—those three great classes of society without whom a country is nowhere in the struggle of life and progress. It does not follow that the nation which takes best care of its diseased or weakly subjects will have the most healthy and prosperous community, and we have perhaps gone a little too far in our study of disease and our outpouring of sentiment over sickness. It should help to rectify the balance if we were to take up as a national duty the study of health and life, and give a little more thought to the production of sound children!

Assuming, then, that we are all agreed as to the necessity of caring for women in childbirth, the next point is, What means do we as a nation provide for meeting this necessity? A woman who takes up the profession of monthly nursing (a most unmeaning and unworthy name for a very responsible and worthy calling, but which I am forced to use for lack of another), must be trained practically as well as theoretically if she is to understand and become skilled in her business. She must be taught what is necessary to be done, why it is necessary, and how to do it. To learn this she must be brought into contact with a sufficiently large number of cases of childbirth in order that she may see a variety of conditions; because it is only in knowing a variety of conditions that the difficulties and dangers concomitant to them can be appreciated; and it is in being able to meet and obviate these dangers and difficulties that the value of a monthly nurse lies. I do not mean that she is necessarily to be trained as a midwife, still less that she should oust the medical man from his proper post; but I do mean that if she has not some knowledge of the mechanism of labour, and the functions of human organs, she is but a broken reed for a helpless young mother to lean on. In normal cases a very little knowledge and a very little skill suffices. Nature and her instincts are strong in a healthy woman and do their own work. But no one can tell beforehand whether the case will be normal or not, and therefore in choosing a nurse the expectant mother wishes to get the most experienced one available. The first thing she asks is "Where were you trained?" or, perhaps, "What sort of training have you had?" and the answer is that the applicant for the post holds her certificate from one of the four large lying-in hospitals of London, one of the smaller maternities, or it may be from a provincial or less well-known institution.

This is for the well-to-do classes of women who have husbands to pay for them. But by far the larger proportion of the mothers of England are not in the happy position of being able to pick and choose a competent person to attend on them, and they have to put up with what they can get. The better-to-do wives in the labouring class, and those of the smaller tradesmen, engage the services of the cheapest medical man in the neighbourhood for the actual delivery, and trust to the more or less incompetent friend or relation for feminine help. In some happier or more fortunate districts private benevolence supplies a midwife or nurse, at a cheap rate, who has been able to get a certain amount of experience, a smattering of knowledge, and a good-natured, overworked doctor to back her up in a difficulty. And for the very poor there is always the workhouse to fall back on in the last emergency—that sole provision which the State makes for the poorest mothers of England.

I am passing no judgment on the State, be it understood; I am only putting the case as it appears on the surface. Lying-in hospitals are not State founded, or State supported, any more than general hospitals. They are private enterprises, either with benevolent intent or for medical schools. And to get the benefit of their help as an "in" or "out-patient," a subscriber's letter is required. There is no such thing as free entrance except for accidents. So much for the provision made by the English people for attendance on its mothers. In our next article we will go back to the training which can be got by those nurses among whom the better-class wives pick and choose.

### Wants and Workers.

Convalescent Home.—Referring to an inquiry in "Notes and Queries" of last week as to a suitable home in which to place a girl in the early stage of phthisis, a correspondent writes: "There is a small home at St. Leonards—Ribbsford House, Chapel Park Road—open from October to May, for young women only, which might be recommended. Inmates are allowed to stay for some months on payment."

### The Book and its Story.

### A PIONEER AMONG WOMEN.

A record of the darker days of the struggle which medical women underwent has been given us by Dr. Elizabeth Blackwell in a little volume she has just published.* The book has a personal as well as an historical interest, and it throws much light on some of the first efforts by means of which the medical profession of our day has been opened to women.

The suggestion of studying medicine was first presented to the authoress, she tells us, in 1845, a suggestion which came from one of her lady friends, who was suffering from a painful disease. The malady was of so distressing a nature that the treatment of it was an added affliction to the sufferer. "You are fond of study; have health and leisure," she once said to Miss Blackwell; "why not study medicine? If I could have been treated by a lady doctor my worst sufferings would have been spared me."

Her friend's words had a great hold on the hearer's mind, and added to this, in a journal of those days, the future physician writes, "I must have something to engross my thoughts, some object in life which will fill the vacuum."

Miss Blackwell was but a young girl when she wrote these words. From all we can gather from her book, she was early imbued with a sense of the responsibility of life, a feeling which was supplemented by an ambition to be a factor in it; to do something, and to be something. Financial difficulties urged on the necessity for work. Her father, a flourishing merchant at Bristol, met with reverse after reverse, and the comfortable, happy home which is pictured to us there had to be given up. In 1832 the Blackwell family emigrated to New York. Six years after this change in their circumstances the bread-winner was snatched from their midst, and, without friends or pecuniary resources, the widow and children were left to face the stern realities of life in a foreign land.

The history of the struggle is told in graphic language by Miss Blackwell's pen, and it was during this time of hardship and privation that the suggestion of a definite line of work was put before her by her friends, as we have described above. As the idea gained force, Miss Blackwell consulted several physicians as to the course she should pursue to enable her to become a doctor. The answers to all her correspondence on this subject were curiously unanimous; all who replied at all, replied to the effect that, though the idea was a good one, its accomplishment was impossible. There was no way, to begin with, of obtaining the necessary education for a woman; and yet this and all other hindering verdicts proved rather a stimulus than a check to the young aspirant's ambition. She writes: "The idea of winning a doctor's degree gradually assumed the aspect of a great moral struggle, and the moral fight had an immense attraction for me," and this moral aspect of the case, Miss Blackwell further tells us, was forced upon her through the existence in New York City, at that time, of a certain Madame Restell, whose illegal practices brought her a great notoriety. The services of this lady were in great demand; she was called "a female physician," a term then used exclusively by those women who carried on this particular occupation.

But strong as was Miss Blackwell's determination to improve this degrading condition of a science which, if carried into legitimate spheres, might become a noble position for women, the difficulties she had to encounter were stupendous. Social dictum was against her on the one hand, and want of means on the other. If public opinion weighed but little with the strong-minded young woman; want of funds was a more serious drawback. How Miss Blackwell overcame the latter obstacle is characteristic of her extraordinary fixity of purpose; the money which was forthcoming from no other source for her intended career, she raised through her own exertions by teaching. In the summer of 1847, with her carefully-hoarded savings, Miss Elizabeth Blackwell resolved to seek an entrance into some medical school. "Philadelphia," she writes, was then "considered the chief seat of medical learning in America." Here her repeated attempts met with refusal after refusal from the medical colleges in the city. The fear of successful rivalry, which at that time often existed in the medical mind, was expressed by the Dean of one of the smaller schools, who frankly replied to her application, "You cannot expect us to furnish you with a stick to break our heads with!"

Some kindly Quaker adviser, whose private lectures Miss Blackwell had been attending, referring to her project, said to her, "Elizabeth, it is no use trying. Thee cannot gain admission to these schools; thee must go to Paris and don *masculine attire* to gain the necessary knowledge." But the suggestion of disguise did not tempt Miss Blackwell, and she persevered in her attempt to gain admission into the smaller schools of the New York State. Amongst these applications was one to the Faculty of the Medical Department of Geneva, a small university town in the States, which opened its doors, in unanimous decree, to the lady student.

The life here, in the winter session of 1847, of one isolated woman among the crowd of male students presents a curious picture, and an unique one in its way. All that we read of that time is of absorbing interest. Here Miss Blackwell's studies was ultimately crowned (in 1849) by a diploma. The admission of a woman for the first time to a complete medica education and full equality in the privileges and the responsibilities of the profession produced a widespread effect in America. For a year or so after her diploma, Miss Blackwell studied in the Maternité Hospital at Paris, and, in London, attended certain lectures at St. Bartholomew's, given by Mr. (afterwards Sir James) Paget. At this hospital, she writes, "Every department was cordially opened to me, *except* the department for female diseases."

Here Miss Elizabeth Blackwell's student career ended, and her professional life commenced. The first seven years of New York life, to which city she repaired in 1851, were years of very difficult, though steady, uphill work. There was no medical companionship for her, and the profession stood aloof. "Society," indeed, in the writer's own words, "was distrustful of the innovation." But her indomitable perseverance, her steady resolve, conquered in the end; and though of this life she gives us too sparing an account, Miss Blackwell closes her narrative whilst she is in the full tide of her medical activity in New York, with a growing private practice, and increasing hospital claims. During the twenty years which followed the graduation of the first woman physician, the public recognition of the justice and advantage of such a measure had steadily grown. Indeed, throughout the Northern States the free and equal entrance of women into the profession of medicine was secured. In Boston, New York, and Philadelphia special medical schools for women were sanctioned by the legislatures, and in some long-established colleges women were received in the ordinary classes.

Dr. Elizabeth Blackwell's story is brightly and pleasantly told. Its value is historical rather than literary, for its writer was a thinker and a worker, rather than an authoress, but as the record of an individual experience of a deeply interesting order, the book will meet many appreciative readers.

* "Pioneer Work in Opening the Medical Profession to Women," Dr. Elizabeth Blackwell. (London: Longmans and Co., 1895.)

### Everybody's Opinion.

[Correspondence on all subjects is invited, but we cannot in any way be responsible for the opinions expressed by our correspondents. No communications can be entertained if the name and address of the correspondent is not given, or unless one side of the paper only be written on.]

### NURSING AT SMALLER PROVINCIAL HOSPITALS.

"AN HONORARY SECRETARY" writes: The efficient nursing of the smaller provincial hospitals is becoming year by year a more difficult matter, and those who are responsible for the management of these institutions will soon have to face this question. I am writing to you in the hope that by ventilating the subject in your valuable paper some remedy may be found. In a hospital of from ten to twenty-five beds, and in which the matron herself acts as nurse, the staff will, perhaps, consist of one nurse who has had three years' regular training and two nurses who have had from one to two years', these latter acting as assistant nurses. There is no necessity that all the nurses employed should have had three years' training; in fact, it is better that they should not, as they act under the "charge nurse," and in consequence occupy a somewhat subordinate position. The applicants for the post of assistant nurse are now, with few exceptions, the halt, the lame, and the blind of the nursing profession. They are usually nurses who have either broken down in health and been obliged to give up the full three years' curriculum, or have left their training school because they were quite unsuited to the work, or have been dismissed for some failure of duty; or they are nurses who have found nursing not quite the "amusement" they expected, and, having no real love for their work, do not care to put up with the discipline of a large hospital. Such nurses are no more wanted in our small hospitals than in our large ones. Almost our only chance of obtaining a good nurse is to engage one who through ill-health alone has failed to complete her training, and who may possibly regain her health under the lighter work of a small hospital. Even if a larger salary than usual be offered, nurses who have gone through a complete three years' course will not apply, as they do not care for a subordinate post at a small hospital, as the holding of such a position might stand in the way of their future career. What then is to be done in order to supply what we so much need, viz., trustworthy nurses who are willing to act in a subordinate position? The only answer I can see to this question is that it must be made possible for nurses to take a period of cottage hospital work as part of their three years' curriculum. I would suggest that each large training school should have affiliated to it a certain number of the smaller provincial hospitals, and that in return for certain payments (which the managers of these hospitals would be willing to make) nurses—say, for the last three or four months of their second year, or for a similar period at the beginning of their third year—should be sent from the large training school to the small hospital. Here they would get change of air and of scene, lighter work, of a somewhat different kind, but still very responsible and useful work. They would have the opportunity, too, of getting an insight into the management and working of a small hospital, at which, or at some similar institution, they may possibly at some future date obtain the post of matron. The matron of the large training school, by making a wise selection of the nurses she told off for this work, might save many a nurse who was getting overworked by the ceaseless toil of a busy and crowded hospital from a breakdown which would jeopardise her chance of ever obtaining her certificate. The advantages would be mutual, and this arrangement would entail no loss of funds to the training school, as the small hospitals would by their payments recoup the former for the loss of the nurse's services during her absence. I merely suggest this arrangement as a possible solution of a very real and increasing difficulty.

### Notes and Queries.

The contents of the Editor's Letter-box have now reached such unwieldy proportions that it has become necessary to establish a hard and fast rule regarding Answers to Correspondents. In future, all questions requiring replies will continue to be answered in this column without any fee. If an answer is required by letter, a fee of half-a-crown must be enclosed with the note containing the enquiry. We are always pleased to help our numerous correspondents to the fullest extent, and we can trust them to sympathise in the overwhelming amount of writing which makes the new rules a necessity. Every communication must be accompanied by the writer's name and address, otherwise it will receive no attention.

### Queries.

(141) *Age Limits.*—I am a private nurse, and find that at the age of thirty-five I cannot join any of the places where employment is found for private nurses. Some of the physicians and surgeons like older nurses, and employ those who have had long experience in private nursing, so the limit seems to me inexplicable. I am a registered nurse and member of the R.B.N.A., and shall be glad of your advice.—*Nurse M.*

(142) *Scotch Schools.*—Can you tell me how I can get a post as charge nurse in a Scotch training school?—*N. M. R.*

(143) *District.*—Do you think the Aberdeen District Nursing Association advertisement is genuine, because it is a long way to go in uncertainty?—*L. O. S.*

(144) *Lepers.*—Will you kindly give me information about the leper settlements? Do they require any more nurses?—*Kathleen.*

(145) *Medical Terms.*—Which is the cheapest dictionary of medical terms which you can recommend?—*Cis.*

(146) *Instruction.*—Where can I get instruction in chiropody for the relief of corns, bunions, and in-growing toe-nail?—*E. F.*

(147) *Training.*—Should I be recognised as a trained nurse after two years in a cottage hospital of ten beds, and easily obtain employment as such?—*Probationer.*

(148) *Brazil.*—Would there be a good opening for a private nurse in or near Rio de Janeiro, working on her own account?—*E. W. R.*

(149) *Training.*—Should I be wise to go for a year to a general hospital? I have had three years' asylum training.—*E. F.*

(150) *Holiday.*—Will you tell me if Aix-la-Chapelle would be a nice place to spend a fortnight in at Easter time, and if there are any cheap pensions there?—*Nurse.*

(151) *Co-operation.*—Can you tell me if there is still a Nurses' Co-operative Company at 18, Featherstone Buildings, Holborn? I have written there, but got no reply?—*R. B.*

### Answers.

(141) *Age Limits (Nurse M.).*—We think you are wrongly informed; at any rate, the Nurses' Co-operation, 8, New Cavendish Street, does not make the age you name the limit. Perhaps your training does not fulfil the requirements insisted on by the rules there. What do you mean by "any of the places"? We should like to know if this refers only to private nursing establishments.

(142) *Scotch Schools (N.M.R.).*—The only plan is to write to the matrons of the Scotch training schools. You will find a list in "Burdett's Hospital Annual." As a rule, the probationers who have received their full training in a hospital are promoted to be staff nurses when vacancies occur.

(143) *District (L.O.S.).*—What do you mean? The Aberdeen District Nursing Association is affiliated with the Scotch Branch of the Queen's Jubilee Institute, and you may think yourself fortunate if your application is favourably considered at headquarters.

(144) *Lepers (Kathleen).*—Do you want to nurse lepers in India, Russia, Ceylon, Robben Island, the Sandwich Islands, or in the North of Europe? Your question is very vague. If you sincerely wish to live a useful and isolated life, why do not you devote your attention to the nursing of your fellow-countrymen and women? In small-pox and fever hospitals the supply of nurses is unequal to the demand, and yet people fail to see that the duty so near home is as urgent as that which attracts them to distant lands to look after lepers, who have, in many cases, all that they require already. At the same time, if you are bent upon joining a leper colony, and will express yourself more definitely, we will answer your question for you with pleasure.

(145) *Medical Terms (Cis).*—The "Nurses' Dictionary," by Honnor Morten, published by the Scientific Press, price 2s.

(146) *Instruction (E. F.).*—Our advertisement columns answer your question. In-growing nail often needs a surgeon's treatment.

(147) *Training (Probationer).*—Such training as you mention would not help you to an engagement in any good nursing institution. Why do you not apply for admission at some large hospital, either in London or the provinces, where the training would be valuable to you afterwards if you are really anxious to take up nursing as a profession? Get "How to Become a Nurse," Scientific Press, 428, Strand, and write to the matrons of the different hospitals.

(148) *Brazil (E. W. R.).*—We do not know of any opening at Rio de Janeiro for a private nurse, and it would be most unwise to attempt such a venture without some certain promise of work from a doctor, or unless you have personal friends in the neighbourhood who would guarantee you a home in case of need.

(149) *Training (E. F.).*—You would be certainly wise to obtain some general training, but you would not be accepted for so short a period at any large London hospital without paying. Try some provincial hospital if you cannot afford a longer time than a year for training and wish for a salary. Get "How to Become a Nurse," Scientific Press, 428, Strand, which will give you full particulars.

(150) *Holiday (Nurse).*—You would find Aix-la-Chapelle rather cold so early in the year. We have no personal knowledge of pensions there. Why not try Dinard, in Brittany? If you feel inclined to follow this suggestion, write again, and you shall be supplied with the address of a pension which can be recommended.

(151) *Co-operation (R. B.).*—You are labouring under some mistake. The address you give was that of a registry now non-existent, and is now that of a wholesale toy dealer and general merchant. The address of the Nurses' Co-operation is 8, New Cavendish Street, W.

### Where to Go.

Grosvenor House.—Women's Total Abstinence Union.—An afternoon meeting for nurses will be held at 2.30 on Friday, February 14th.

Westminster Town Hall.—A concert in aid of the Royal Hospital for Children and Women will be given at half-past eight on Tuesday, February 25th, by the Children's Orchestra.

Arlington House, Uxbridge Road.—The opening by the Countess of Chesterfield of this branch of the St. John's Hospital for Diseases of the Skin will take place at 3 30 on Monday, February 17th.

Royal British Nurses' Association.—The third sessional lecture of the season will be given on Friday, February 21st, at 8 p.m., at the offices of the association, 17, Old Cavendish Street, by Dr. Wethered, the subject being "Bacteriology," with demonstrations. Members are admitted free, the general public on payment of 1s.

The Grafton Gallery (Loan collection of modern pictures of the Dutch and Barbizon schools, February to March 31st, 1896).—Though the painters whose works are represented now at the Grafton Gallery are placed under the grouping of a "school," the men who composed it were ignorant of such classification. To speak more literally, the French and Dutch romanticists of this century formed a school only in the sense that they emancipated themselves from the trammels of conventionality and chose to think for them selves, and to see for themselves. In of the works now gathered together, one cannot fail to notice this fact. Millet thought for himself; Corot thought for himself; and in the expression, too, of their art Israels, Michel, and all the so-called "Barbizon" school were alike individual. In the work of these men you look in vain for trickery, for affectation, for pettiness, for imitation; it was perhaps their intentions, their intellectuality, more than their methods (unique in each case), which bound these artists together in their bond of fellowship. This "Barbizon School" was so named, as many of us are aware, after a little village in the Forest of Fontainebleau, where, for the most part, the immortal works now collected together were produced. Whilst they lived and laboured, the paintings of Millet and Corot, the master minds of the group, sufficed only to support their designers, and to produce the bare necessities of life. For instance, £20 was the purchase-money of the "Angelus" at the time of its production, whilst its last sale-money realised £25,000. And Corot, too—his life was little but a struggle against want; but by his dying words, which have been handed down to us, we gather he had neither lost hope nor realised the bitterness of a want of fuller contemporary appreciation. "When the spring comes," he said, "I will paint a beautiful picture. I see a sky full of roses." From generalising on the men, we must glance at their work, without actually specifying it, for to specify one would be to specify all, each work which is hung here requiring an individual study, which space prevents. One picture can barely be picked out more than another, each in the collection, perhaps without any exception, being a masterpiece in itself. The directors of the Galleries are to be much congratulated on the excellent manner in which they have arranged the collection; nothing throughout could be improved upon. The Octagon Room is hung with works of Jozef Israels, the veteran Dutchman; he worked at Amsterdam and at Paris, but his true masters were to be found in Rembrandt and in Millet. Israels has never been better shown in this country, strange to say, than in the limited space of the First Room at the Grafton. A sketch of the master, by his own pencil, epitomises to us the biography of the man. Calm, sympathetic, and direct, his face reveals his mind as his mind is revealed in his works. These paintings show a quick sympathy with humanity. Israels was the great painter of Dutch national life, the pathos of which is powerfully expressed through the medium of his brush. Passing into the adjoining room, we come first upon Corot and then upon Millet. These drawings, although so different, marvellously suggestive of light and space and atmosphere. Here, among the Millets, is the "Angelus" (a reduction from the larger original), that pathetic pastoral known so well to us all as to require no criticism here. In fact, all through the galleries the pictures of Millet, Corot, Dupré, Diaz, Maris, and Rousseau disarm one's critical faculties by their individual beauty. In the end gallery two hundred black and white drawings, representativ of London life, its streets, its barracks, its Senate, and hospitals, are exhibited, from Paul Renouard's inimitable pencil.

### For Reading to the Sick.

### RESPONSIBILITY.

**Motto.**

Our responsibility as Christians corresponds with the grandeur of the truth which is placed within our reach.—*Bishop Wescott.*

**Verses.**

God bends from out the deep and says:
"I gave thee the great gift of Life;
Was't thou not called in many ways?
Are not my earth and heaven at strife?
I gave thee of my seed to sow—
Bringest thou Me My hundredfold?"
Can I look up with face aglow,
And answer, "Father, here is gold"?
—*Lowell.*

What is it then to me
If others are inquisitive to see?
Why should I quit my place to go and ask
If other men are working at their task?
Leave my own buried roots, to go
And see that brother plants shall grow;
And turn away from Thee, O Thou most Holy Light,
To look if other orbs their orbits keep aright
Around their proper sun,
Deserting Thee, and being undone? —*Clough.*

Man who man would be,
Must rule the empire of himself; in it
Must be supreme, establishing his throne
On vanquished will, quelling the anarchy
Of hopes and fears, being himself alone. —*Shelley.*

**Reading.**

'Tis in the advance of individual minds that the slow crowd should ground their expectation, eventually to follow. —*Browning.*

Nothing can alter the Responsility which is laid upon each soul.—*Wescott.*

Each of us brings with him an element, more or less important, of the life of Humanity to come.—*Mazzani.*

Are not great men the models of nations?—*Lytton.*

It is great folly not to part with your own faults, which is possible, but to try instead to escape from other people's faults, which is impossible.—*Marcus Aurelius.*

For everyone of us may, if he will, do something, be it much or little, towards making that which will be the history of our generation; and the abiding worth of whatever he can do will depend, perhaps, mainly on his just discernment of the chief issues that are being either decided or kept open in his day; on his correcting in his own mind the misplaced emphasis of common talk and controversy; on his throwing whatever strength he has into the real, and not the merely apparent crisis of the perpetual conflict between truth and error, between good and evil, or between the better and the less good. It is a safeguard against all misdirection of vehemence and solicitude, it may help us to give to the real task of our day whatever energy or influence we have to dedicate, if, from time to time, looking back to past ages of especial trial and confusion, we single out in the *mêlée* of the fight those whom time, the great arbiter of all blunders, has approved as the men who were not misled; who saw for what they must contend, and held to that; who were strong enough to do without the encouragement of easy triumphs, fighting neither with small nor great, but only with the antagonist whose onset was making for the true centre of their position—the men who not only meant well, but went right.—*Dean Francis Paget.*

